# Supplementary material for: rMSIcleanup: an open-source tool for matrix-related peak annotation in mass spectrometry imaging and its application to silver-assisted laser desorption/ionization
Source: J Cheminform. 2020 Jul 22;12:45. doi: 10.1186/s13321-020-00449-0 (PMC7374922; doi:10.1186/s13321-020-00449-0)
Supplement: Supplementary file 1 — Additional file 1. Additional Figures and Tables. [file 13321_2020_449_MOESM1_ESM.pdf]

ADDITIONAL FILE 1

A. Visual Report

```
##########################################################################
– Package Version: 0.1.1
– Time: 2019–07–23 13:39:26
##########################################################################
IMAGE INFORMATION
– Peak matrix: 2016_06_01_Brain_Control–Ag.tar
– Full spectrum: NULL
– Number of peaks: 135
– Number of pixels: 10010
– Mass Range: [ 81.0431355625551 , 1002.15406104405 ]
##########################################################################
MATRIX INFORMATION
– Matrix formula: Ag1; Ag1F1; Ag1Cl1; Ag1Br1; Ag1I1; Ag1H1; Ag1H2; Ag1He1; Ag1N1O3; Ag1Th2; Ag1F2; Ag1B1F4; Ag1C27H[illegible]
Ag1C26H54O1; Ag1C28H58O1; Ag1C30H62O1; Ag1C26H52O2; Ag1C30H60O2
– Add list: F1; Cl1; Br1; I1; H1; H2; He1; N1O3; Th2; F2; B1F4; C27H56; C29H60; C31H64; C26H54O1; C28H58O1; C30H62O1; [illegible]
– Substract list: NULL
– Maximum cluster multiplication: 10
– Base forms: Ag1; Ag1H1; Ag1H2; Ag1He1; Ag1F1; Ag1Cl1; Ag1F2; Ag1N1O3; Ag1Br1; Ag1B1F4; Ag2; Ag2H2; Ag2H4; Ag2He2
Ag2F4; Ag3; Ag3H3; Ag3H6; Ag3He3; Ag2N2O6; Ag2Br2; Ag3F3; Ag2B2F8; Ag3Cl3; Ag4; Ag4H4; Ag3F6; Ag4H8; Ag4He4; Ag2I2
Ag1C26H52O2; Ag4F4; Ag3N3O9; Ag1C29H60; Ag1C28H58O1; Ag5; Ag5H5; Ag1C31H64; Ag5H10; Ag1C30H62O1; Ag5He5; Ag
Ag4F8; Ag3B3F12; Ag5F5; Ag6; Ag6H6; Ag6H12; Ag6He6; Ag4N4O12; Ag3I3; Ag5Cl5; Ag5F10; Ag4Br4; Ag7; Ag7H7; Ag6F6; Ag[illegible]
Ag5N5O15; Ag6Cl6; Ag8; Ag8H8; Ag6F12; Ag8H16; Ag7F7; Ag8He8; Ag5Br5; Ag4I4; Ag9; Ag5B5F20; Ag9H9; Ag2C54H112; Ag2[illegible]
Ag9He9; Ag2C52H104O4; Ag8F8; Ag6N6O18; Ag7F14; Ag2C58H120; Ag2C56H116O2; Ag10; Ag10H10; Ag2C62H128; Ag10H20
Ag2C60H120O4; Ag9F9; Ag8Cl8; Ag2Th4; Ag8F16; Ag6B6F24; Ag5I5; Ag7N7O21; Ag10F10; Ag9Cl9; Ag7Br7; Ag9F18; Ag8N8O2[illegible]
Ag10F20; Ag3C81H168; Ag3C78H162O3; Ag8Br8; Ag3C78H156O6; Ag9N9O27; Ag3C87H180; Ag8B8F32; Ag3C84H174O3; Ag3[illegible]
Ag3C90H180O6; Ag10N10O30; Ag3Th6; Ag9B9F36; Ag10Br10; Ag8I8; Ag10B10F40; Ag4C108H224; Ag4C104H216O4; Ag4C104[illegible]
Ag9I9; Ag4C124H256; Ag4C120H248O4; Ag4C120H240O8; Ag4Th8; Ag10I10; Ag5C135H280; Ag5C130H270O5; Ag5C130H260[illegible]
Ag5C155H320; Ag5C150H310O5; Ag5C150H300O10; Ag5Th10; Ag6C162H336; Ag6C156H324O6; Ag6C156H312O12; Ag6C174
Ag6C180H372O6; Ag6C180H360O12; Ag7C189H392; Ag7C182H378O7; Ag6Th12; Ag7C182H364O14; Ag7C203H420; Ag7C196
Ag8C216H448; Ag8C208H432O8; Ag7C210H420O14; Ag7Th14; Ag8C208H416O16; Ag8C232H480; Ag8C224H464O8; Ag8C248
Ag9C234H486O9; Ag8C240H480O16; Ag9C234H468O18; Ag8Th16; Ag9C261H540; Ag9C252H522O9; Ag10C270H560; Ag9C27
Ag10C260H520O20; Ag9C270H540O18; Ag9Th18; Ag10C290H600; Ag10C280H580O10; Ag10C310H640; Ag10C300H620O10; [illegible]
##########################################################################
PROCESSING INFORMATION
– S1 threshold: 0.8
– S2 threshold: 0.8
– Similarity method: euclidean
– Magnitude of interest: intensity
– Tolerance mode: scans
– Tolerance scans: 4
##########################################################################
```

Additional file 1: Fig. S1 Initial summary of the results including main metrics of the images, the chemical formulae, the potential cluster adduct and neutral losses.

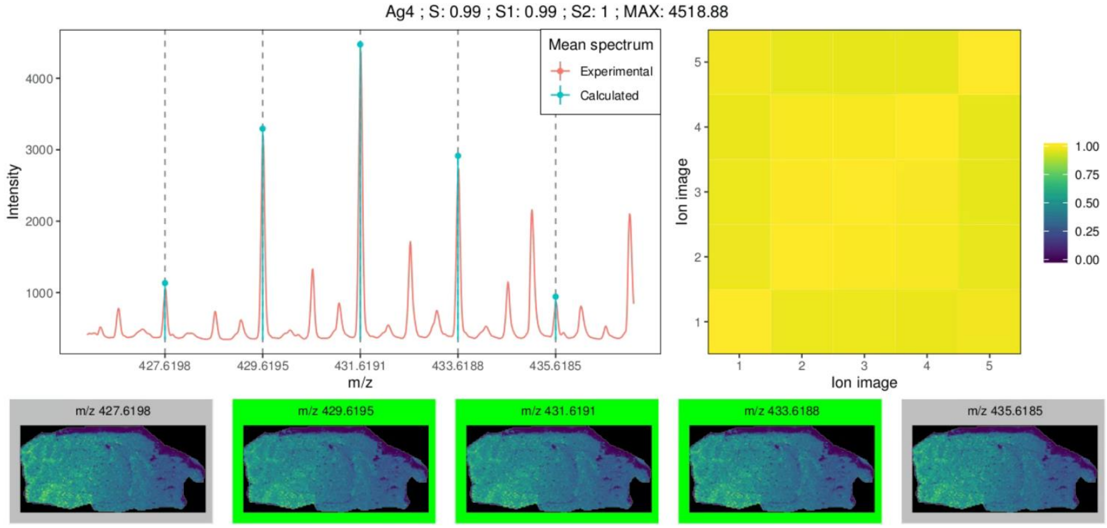

Additional file 1: Fig. S2 Visual report for cluster Ag4 in Dataset 7. The report includes the comparison between experimental and calculated peaks, the correlation map and all ionic images. The ionic images with a green border

are tagged as silver-related. The ionic images with a grey border are not found in the peak list provided and are thus not classified.

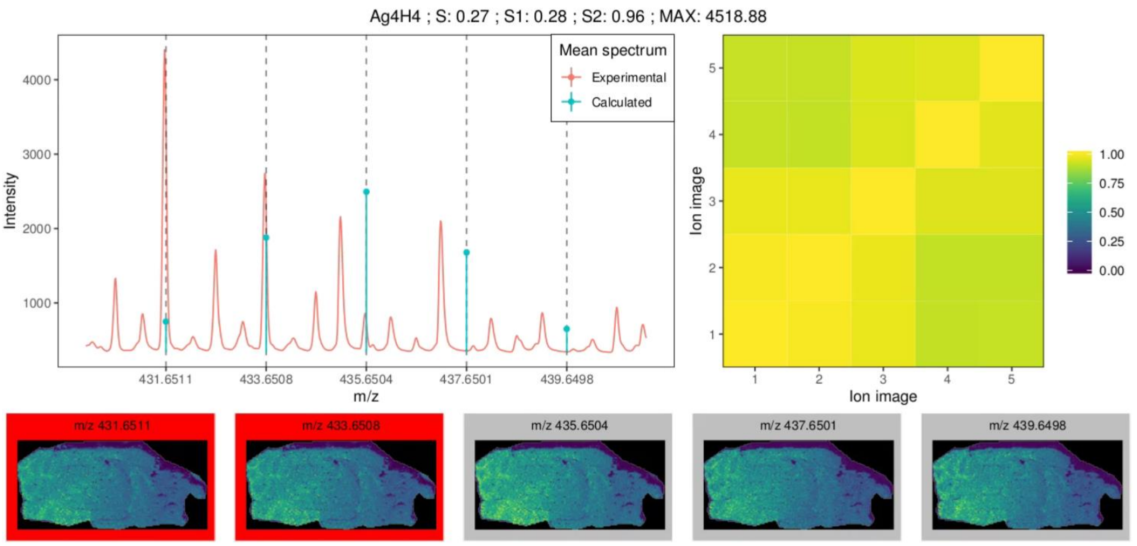

Additional file 1: Fig. S3 Visual report for cluster Ag4H4 in Dataset 7. The report includes the comparison between experimental and calculated peaks, the correlation map and all ionic images. The ionic images with a red border are tagged as not silver-related. The ionic images with a grey border are not found in the peak list provided and are thus not classified.

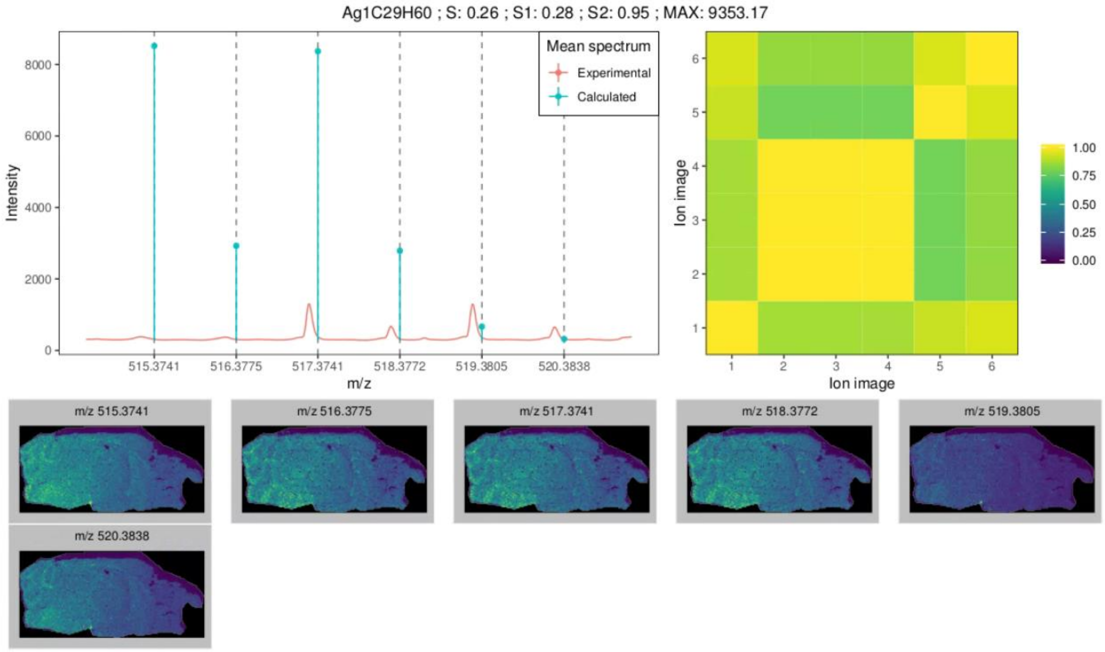

Additional file 1: Fig. S4 Visual report for cluster AgC29H60 in Dataset 7. The report includes the comparison between experimental and calculated peaks, the correlation map and all ionic images. The ionic images with a grey border are not found in the peak list provided and are thus not classified.

B. Table of cluster numbers

1	Ag_1	28	Ag_4Cl_4	55	Ag_7F_7
2	Ag_2	29	Ag_6He_6	56	$C_{60}H_{124}O_2 + Ag_2$

3	Ag_5	30	Ag_3Br_3	57	$Ag_5B_5F_{20}$
4	Ag_7	31	Ag_4H_4	58	Ag_8Cl_8
5	Ag_4	32	$Ag_1N_1O_3$	59	Ag_8H_8
6	Ag_9	33	$C_{26}H_{52}O_2 + Ag$	60	$C_{26}H_{54}O_1 + Ag_1$
7	Ag_3	34	$C_{29}H_{60} + Ag$	61	$C_{60}H_{120}O_4 + Ag_2$
8	$C_{28}H_{58}O_1 + Ag$	35	Ag_5F_{10}	62	Ag_9H_9
9	Ag_8	36	Ag_2H_4	63	$Ag_4B_4F_{16}$
10	Ag_6	37	Ag_5Cl_5	64	Ag_2F_4
11	Ag_3Cl_3	38	Ag_1I_1	65	Ag_5He_5
12	Ag_2TH_4	39	Ag_9He_9	66	$Ag_7N_7O_{21}$
13	Ag_2H_2	40	Ag_6F_{12}	67	Ag_9F_9
14	$C_{30}H_{60}O_2 + Ag$	41	Ag_1Cl_1	68	Ag_8F_{16}
15	Ag_6F_6	42	$C_{54}H_{112} + Ag_2$	69	$Ag_{10}He_{10}$
16	Ag_7H_7	43	Ag_3H_6	70	$Ag_4N_4O_{12}$
17	Ag_1F_2	44	Ag_7He_7	71	$Ag_6B_6F_{24}$
18	Ag_3I_3	45	$C_{52}H_{108}O_2 + Ag_2$	72	Ag_7F_{14}
19	Ag_1H_2	46	Ag_6H_{12}	73	$C_{52}H_{104}O_4 + Ag_2$
20	Ag_5F_5	47	$C_{62}H_{128} + Ag_2$	74	$C_{56}H_{116}\ O_2 + Ag_2$
21	Ag_1He_1	48	$Ag_1B_1F_4$	75	Ag_9H_{18}
22	Ag_6Cl_6	49	Ag_6H_6	76	Ag_8F_8
23	Ag_{10}	50	Ag_7H_{14}	77	$C_{58}H_{120} + Ag_2$
24	Ag_4Br_4	51	$Ag_5N_5O_{15}$	78	Ag_6Br_6
25	Ag_3F_6	52	Ag_5I_5	79	$Ag_{10}H_{10}$
26	Ag_4I_4	53	Ag_5H_{10}	80	$Ag_{10}H_{20}$
27	Ag_8He_8	54	Ag_8H_{16}	81	Ag_7Cl_7

Additional file 1: Table S1. Cluster numbers used in Figure 1C in decreasing order of mean S1·S2 performance

C. Example clusters

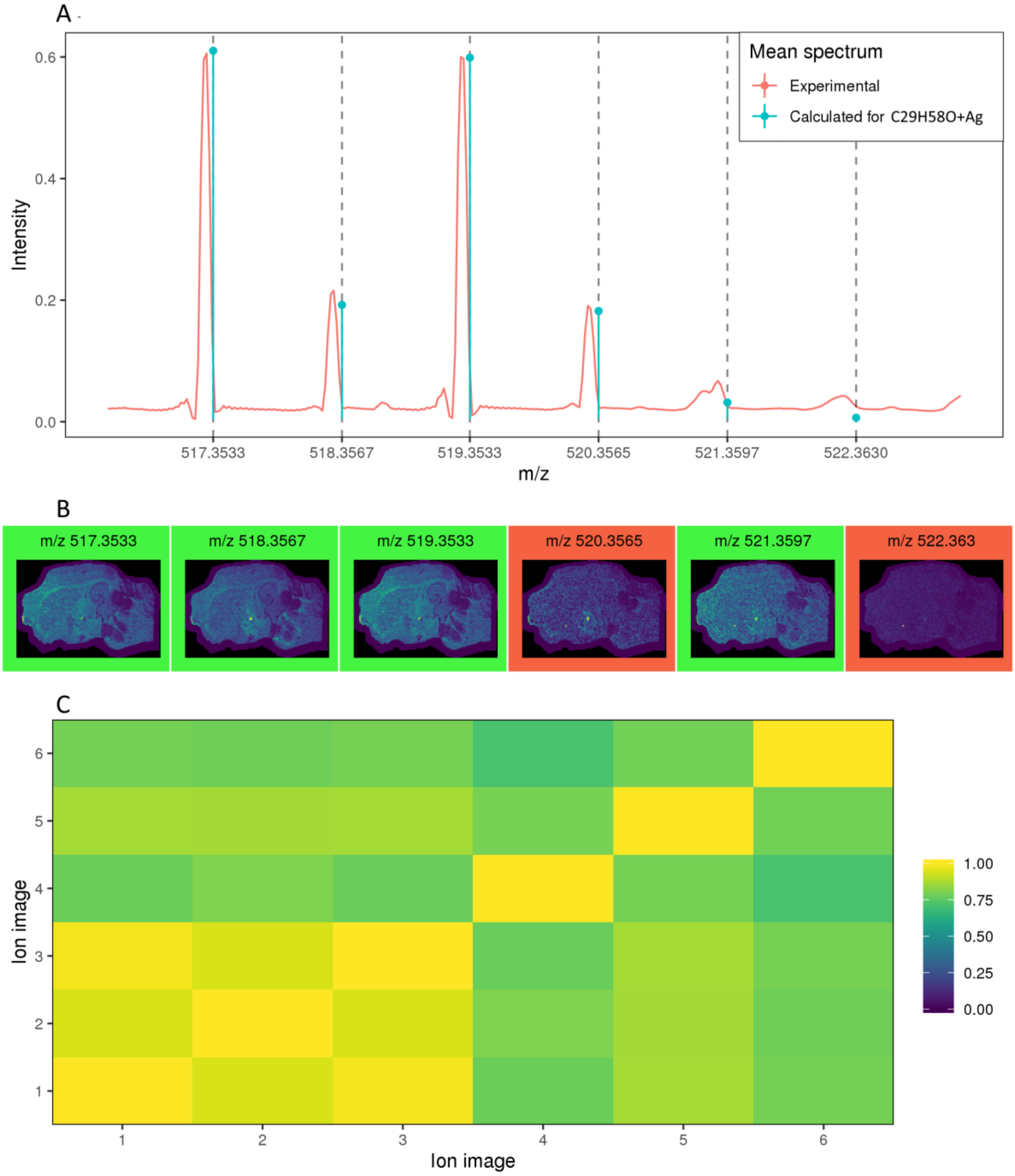

Additional file 1: Fig. S5 Classification results of cluster $C_{28}H_{58}O + Ag$ in Dataset 4. (A) Comparison between the mean experimental spectra and the theoretical pattern. (B) Spatial distributions of the experimental cluster peaks. (C) Correlation matrix between the experimental ionic images of the cluster. The cluster is misclassified as silver-related (false positive). Further study and annotation of these peaks would be needed to assess if the compound is indeed present in the sample implying that this specific compound should not be included in the "ground truth" as a negative class. Nevertheless, the constant and notable mass error between experimental and theoretical peaks allows us to infer that the experimental pattern might be due to a different compound. Adjusting the mass tolerance of the algorithm would get rid of these false positives.

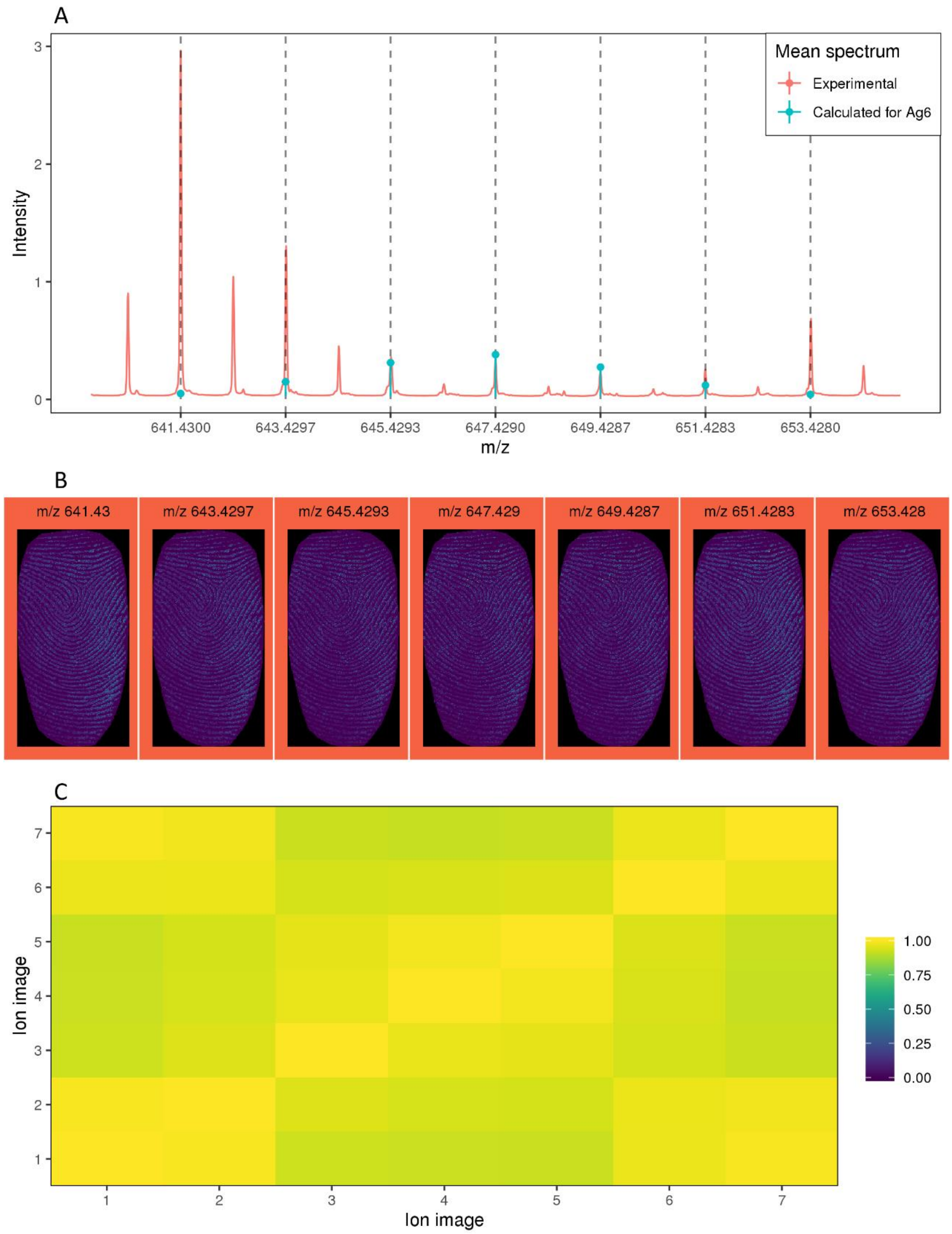

Additional file 1: Fig. S6 Classification results of cluster Ag_6 in Dataset 12. (A) Comparison between the mean experimental spectra and the theoretical pattern. (B) Spatial distributions of the experimental cluster peaks. (C) Correlation matrix between the experimental ionic images of the cluster. The cluster is misclassified as not silver-related (false negative). Like the example in Figure 2, peaks m/z 641.43, m/z 643.43 and m/z 653.43 clearly suffer from overlapping. Nevertheless, due to the high homogeneity of the fingerprint sample, the morphological correlation between the overlapped and the non-overlapped ions is relatively high. The overlapping detection algorithm fails to detect the overlapped peaks.

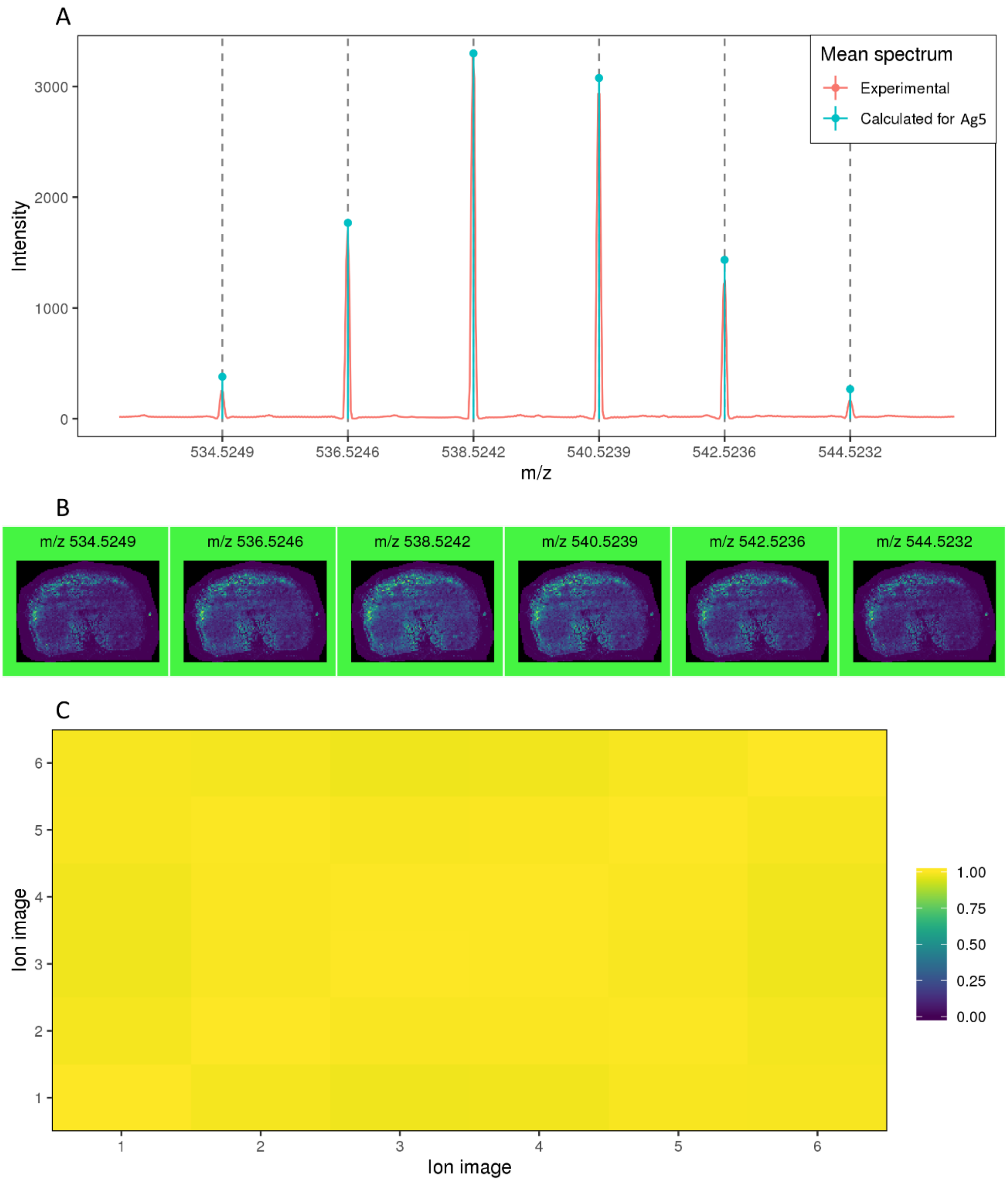

Additional file 1: Fig. S7 Classification results of cluster Ag_5 *in Dataset 3. (A) Comparison between the mean experimental spectra and the theoretical pattern. (B) Spatial distributions of the experimental cluster peaks. (C) Correlation matrix between the experimental ionic images of the cluster. The cluster is correctly classified as silver-related (true positive).*

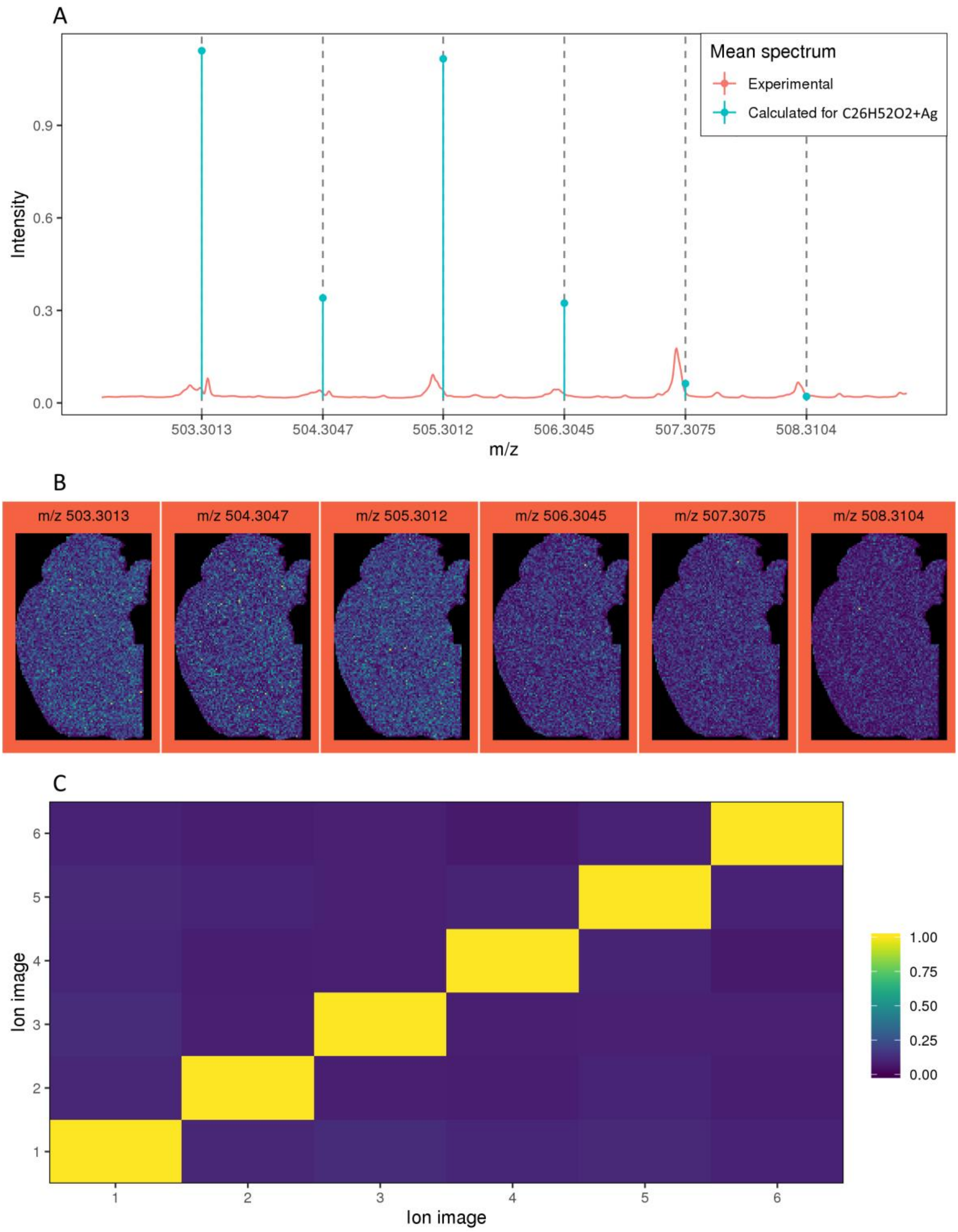

Additional file 1: Fig. S8 Classification results of cluster $C_{26}H_{52}O_2 + Ag$ in Dataset 11. (A) Comparison between the mean experimental spectra and the theoretical pattern. (B) Spatial distributions of the experimental cluster peaks. (C) Correlation matrix between the experimental ionic images of the cluster. The cluster is correctly classified as not silver-related (false positive).

D. Effects of overlapping peak detection

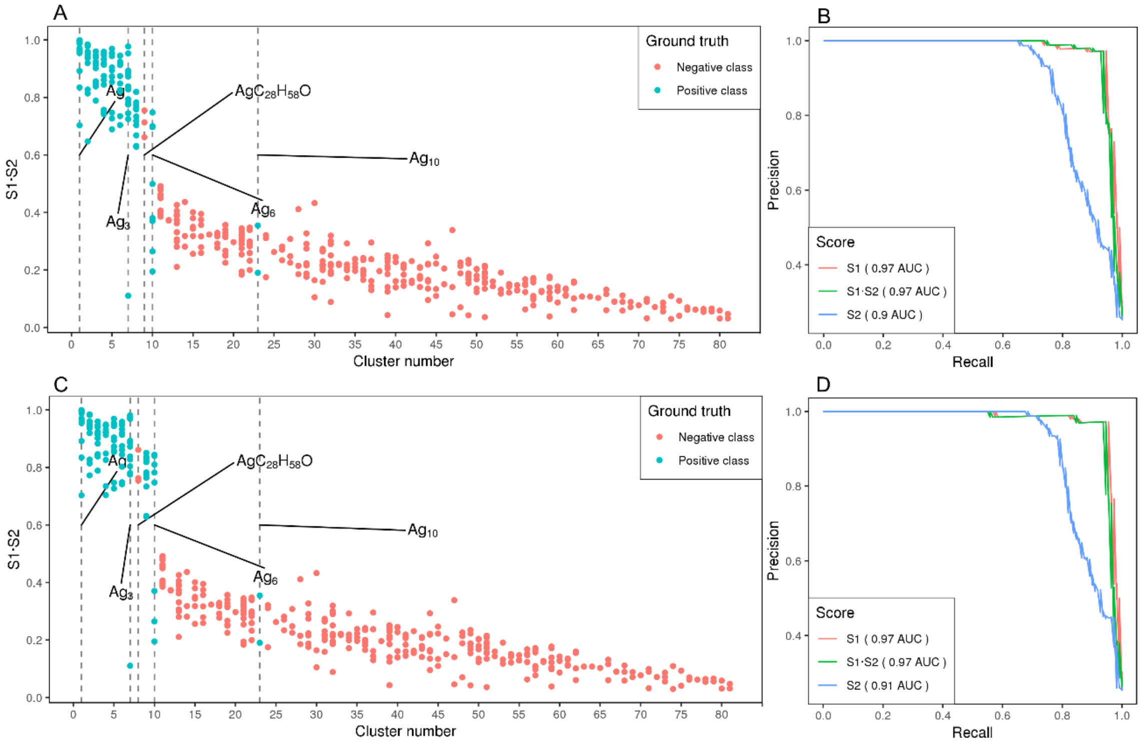

Additional file 1: Fig. S9 Similarity score S1·S2 vs. Cluster number and Precision vs. Recall curves with overlapping peak detection disabled or enabled. (A) & (B) Overlapping peak detection disabled. Multiple Ag6 clusters receive a low score and are thus misclassified as not Ag-related. (C) & (D) Overlapping peak detection enabled. The number of misclassified Ag6 clusters is considerably reduced. Additionally, the gap between the positive and negative class is now bigger leading to a more robust thresholding.

E. Complete exploratory analysis using PCA

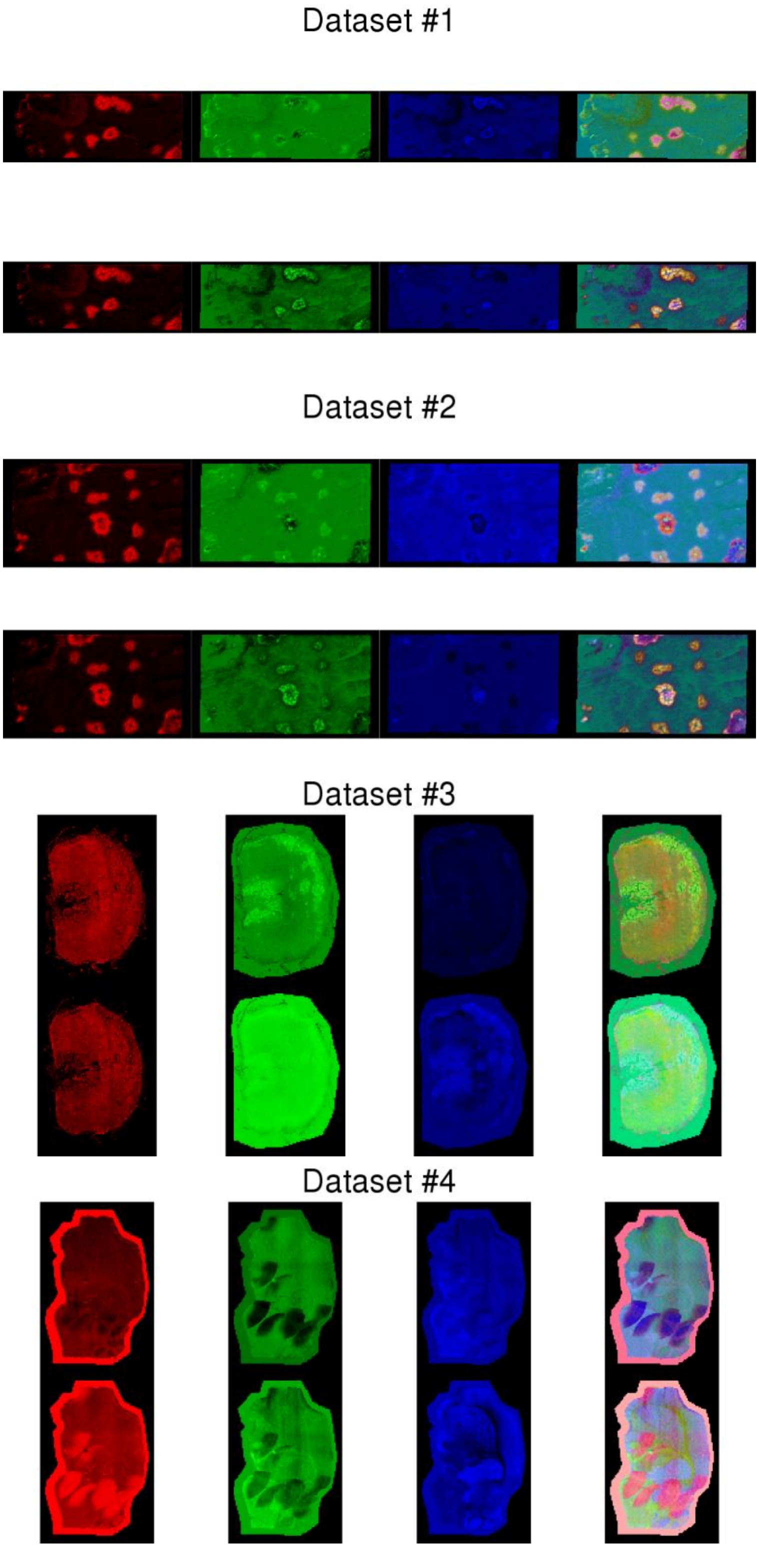

Additional file 1: Fig. S10 Exploratory analysis with PCA before (top row) and after (bottom row) removing matrix-related peaks for Datasets 1-4. Red, green and blue are used to represent the spatial distribution of PC1, PC2 and PC3, respectively. The last column uses the Red Green Blue colour model (RGB) to represent the first three principal components in a single image.

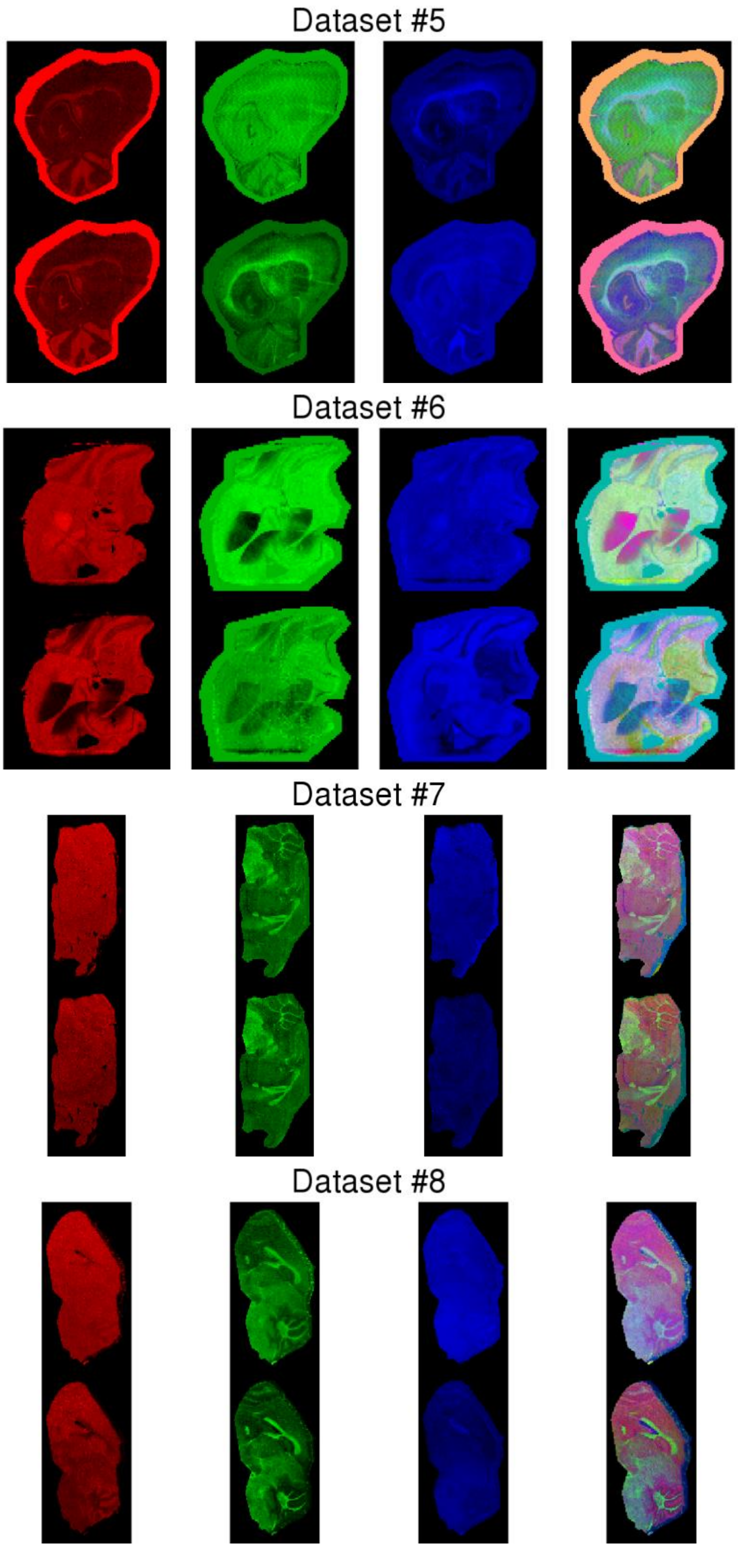

Additional file 1: Fig. S11 Exploratory analysis with PCA before (top row) and after (bottom row) removing matrix-related peaks for Datasets 5-8. Red, green and blue are used to represent the spatial distribution of PC1, PC2 and PC3, respectively. The last column uses the Red Green Blue colour model (RGB) to represent the first three principal components in a single image.

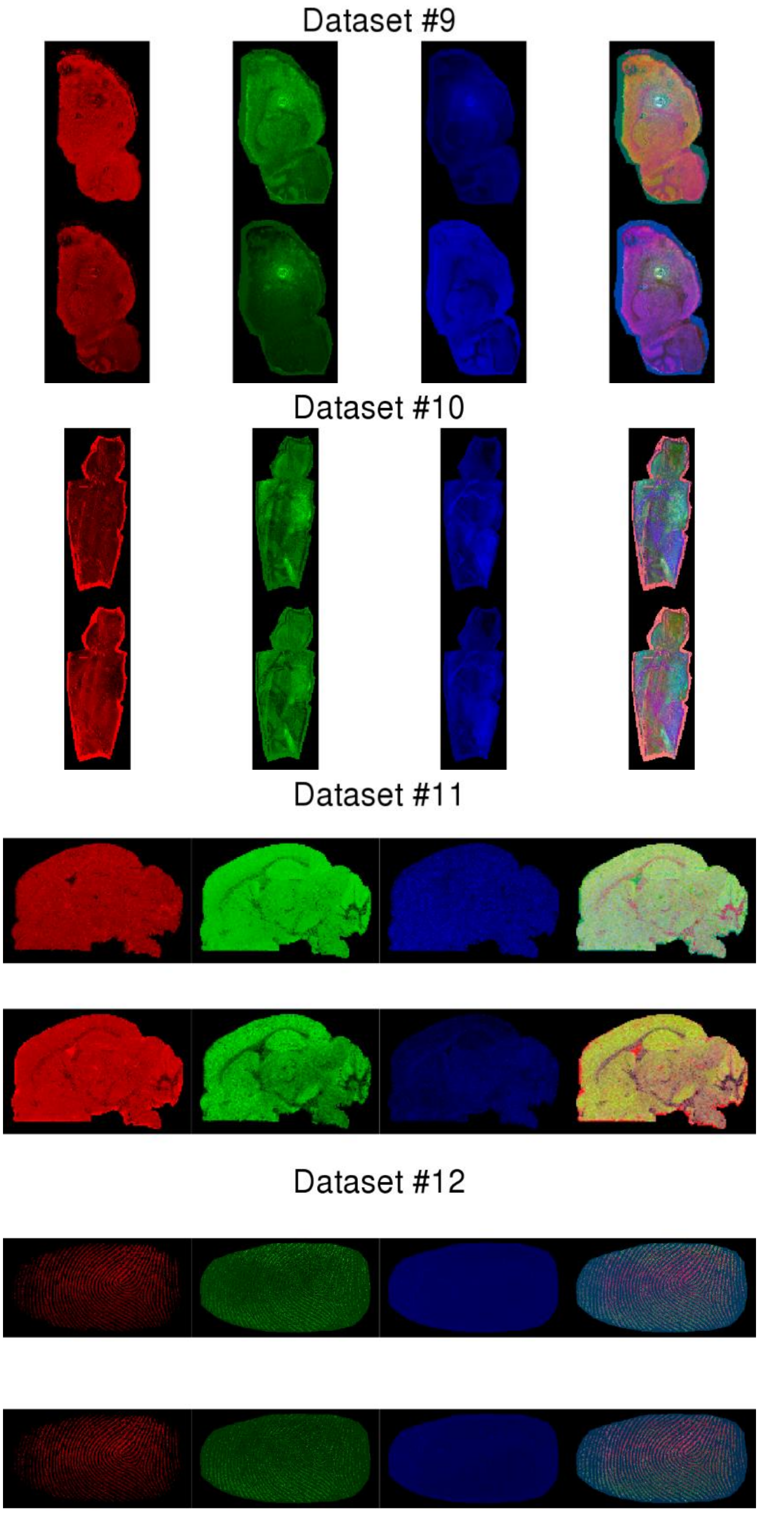

Additional file 1: Fig. S12 Exploratory analysis with PCA before (top row) and after (bottom row) removing matrix-related peaks for Datasets 9-12. Red, green and blue are used to represent the spatial distribution of PC1, PC2 and PC3, respectively. The last column uses the Red Green Blue colour model (RGB) to represent the first three principal components in a single image.

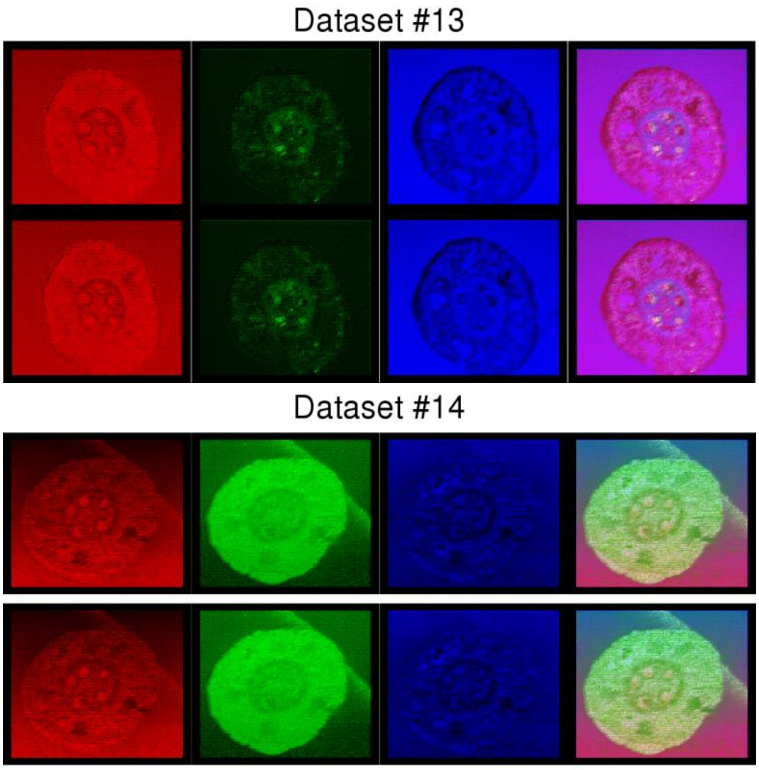

Additional file 1: Fig. S13 Exploratory analysis with PCA before (top row) and after (bottom row) removing matrix-related peaks for Datasets 13-14. Red, green and blue are used to represent the spatial distribution of PC1, PC2 and PC3, respectively. The last column uses the Red Green Blue colour model (RGB) to represent the first three principal components in a single image.

Dataset	# Peaks	# Ag_n^+ peaks	Reduction ratio	TIC % (Ag_n^+ peaks)
1	1164	53	4.55%	31.78%
2	1164	51	4.38%	34.47%
3	381	46	12.07%	50.41%
4	621	55	8.86%	55.63%
5	625	45	7.2%	45.59%
6	585	41	7%	52.89%
7	135	39	28.89%	50.65%
8	135	39	28.89%	54.88%
9	174	43	24.71%	57.92%
10	174	43	24.71%	57.39%
11	1028	51	4.96%	49.99%
12	544	57	10.48%	32.75%
13	693	6	0.87%	2.71%
14	488	2	0.41%	0.07%

Additional file 1: Table S2 Number of peaks, number of annotated Ag_n^+ peaks, reduction ratio and percentage of the Total Ion Count (TIC) accounted by Ag_n^+ peaks for all datasets.

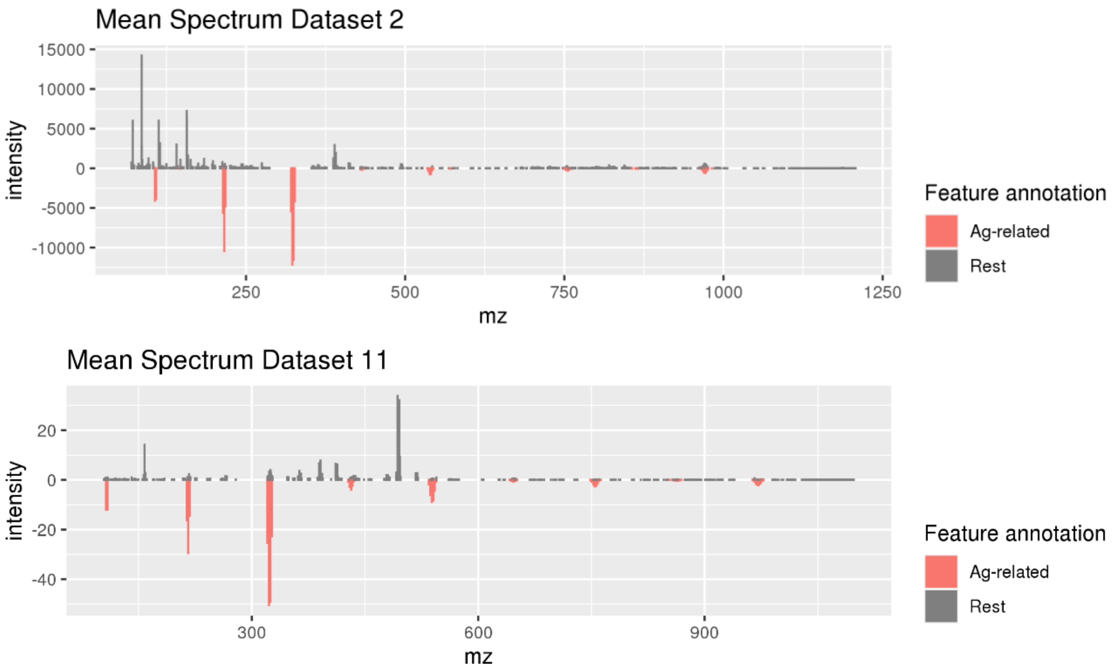

Additional file 1: Fig. S14 Mean spectra of Datasets 2 and 11. Red highlights Ag-related peaks.

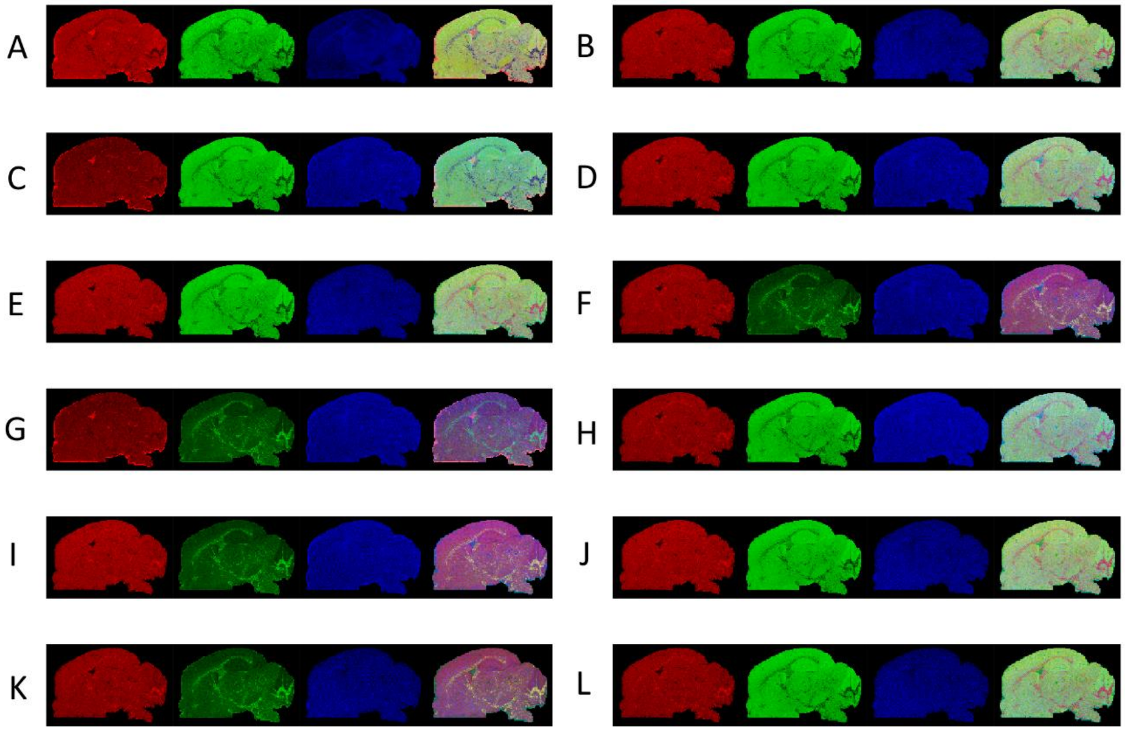

Additional file 1: Fig. S15 Exploratory analysis with PCA for Dataset 11. Matching the same number of features before and after removing matrix-related peaks. The Red, green and blue are used to represent the spatial distribution of PC1, PC2 and PC3, respectively. The last column uses the Red Green Blue colour model (RGB) to represent the first three principal components in a single image. (A) After removing matrix-related peaks. Containing 977 features. (B) Before removing matrix-related peaks. Selecting the 977 most intense features. (C-L) Before removing matrix-related peaks. Selecting 977 features randomly 10 times.

F. Performace comparison to blank subtraction

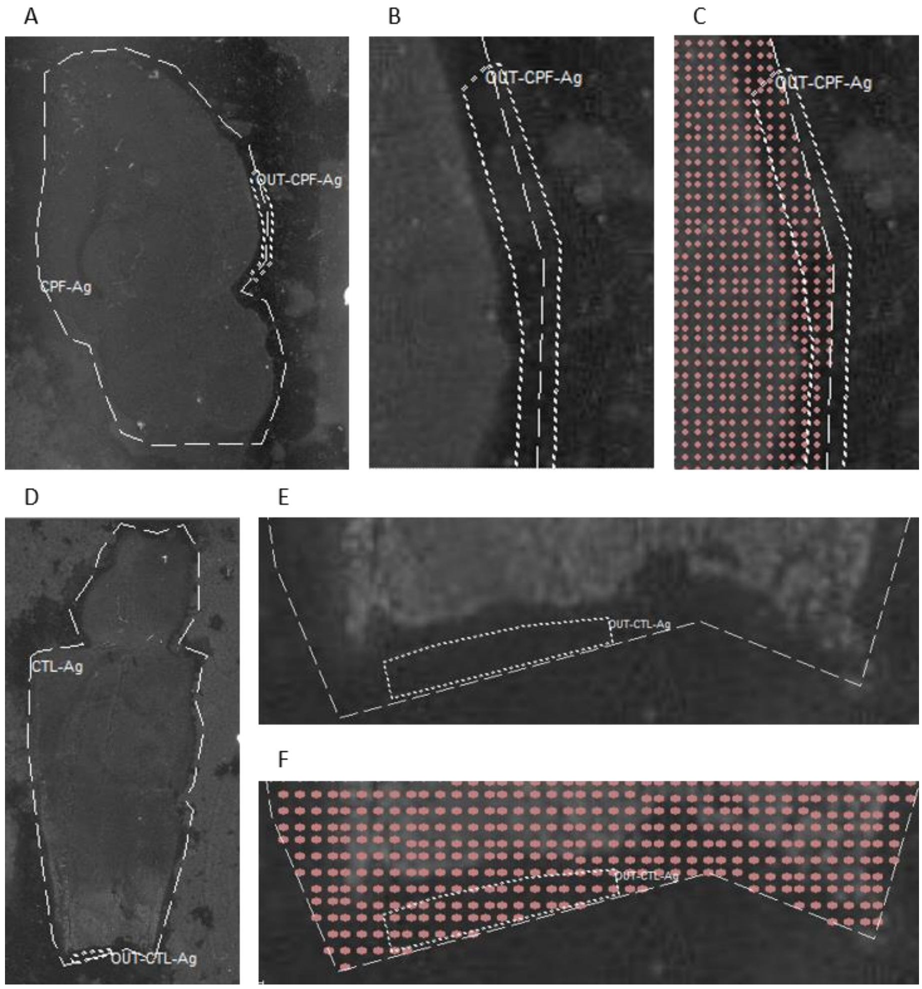

Additional file 1: Fig. S16 Regions Of Interest (ROI) outside of sample used to perform blank substraction. (A) Optical image of the brain slice used for Dataset 9. (B) Zoom-in of the off-sample ROI (C) Laser spots detail (D) Optical image of the brain slice used for Dataset 10. (E) Zoom-in of the off-sample ROI (F) Laser spots detail

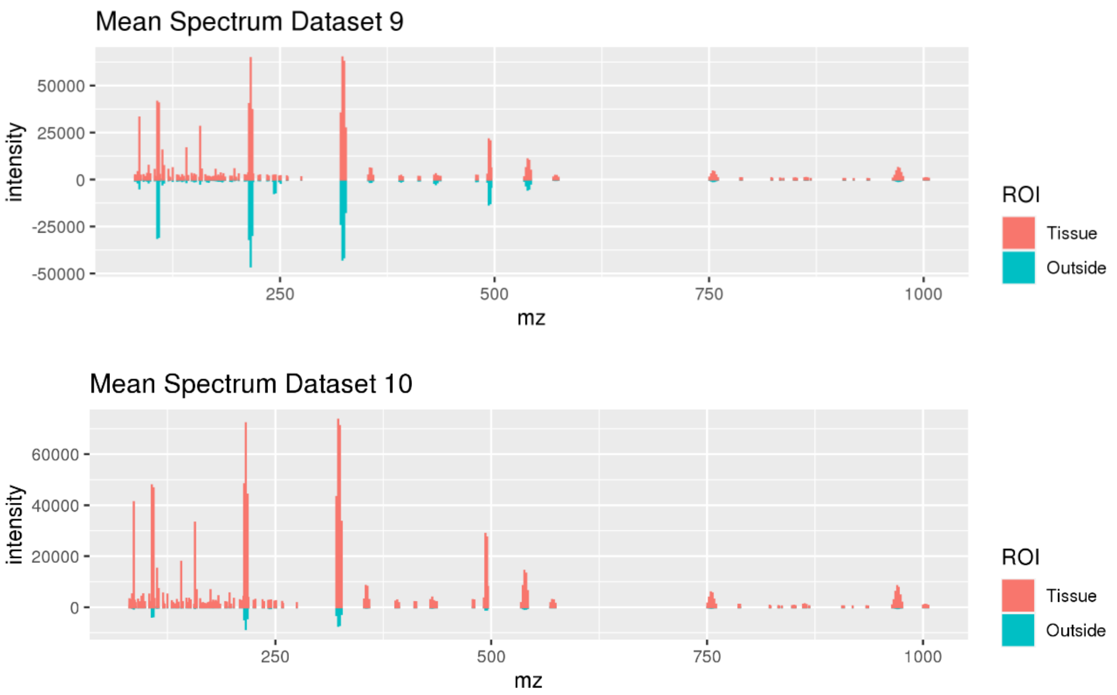

Additional file 1: Fig. S17 Mean spectra comparison between on-sample ROI (red) and off-sample ROI (blue) for Dataset 9 (top) and Dataset 10 (bottom). The out-sample spectra are considerably less intense and it is apparent that there are signals other than Ag-related signals.

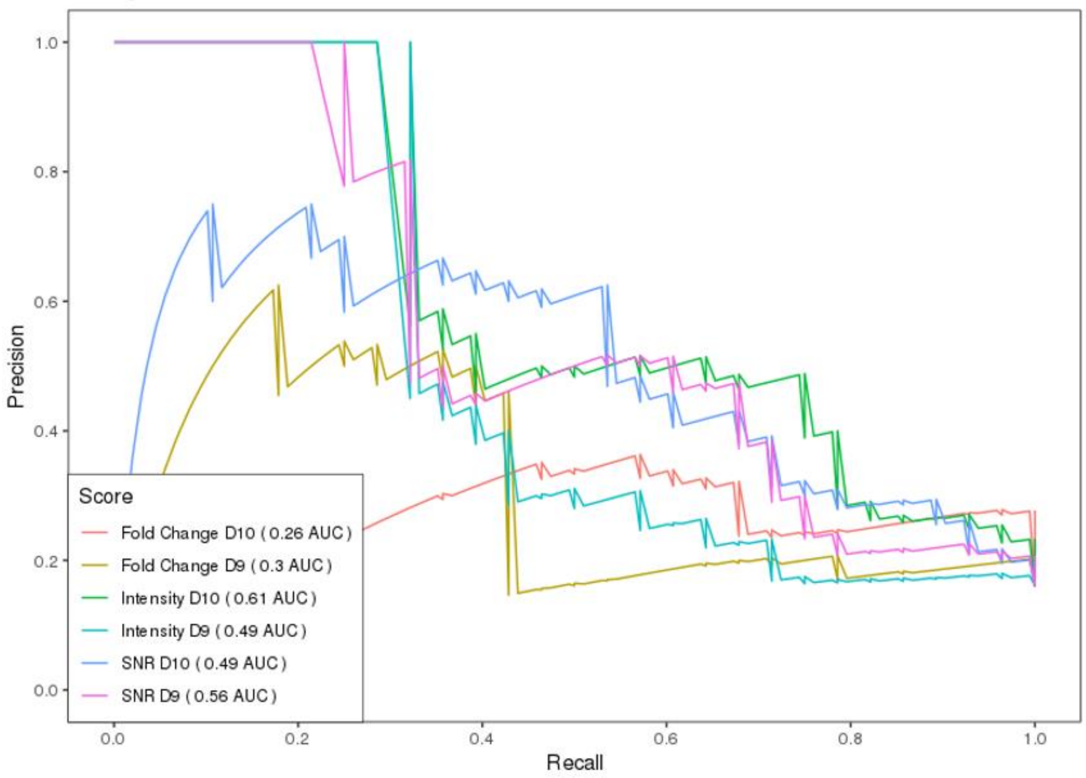

Additional file 1: Fig. S18 Precision and recall (PR) curve for background subtraction using three different metrics (Fold Change, Intensity and SNR) for Datasets 9 and 10. SNR and intensity are better classifiers than Fold Change as they report considerably higher area under the curve. The highest AUC of 0.61 is reported for Dataset 10 using Intensity as the classification metric.
